# Performance of Choline Geranate Deep Eutectic Solvent as Transdermal Permeation Enhancer: An In Vitro Skin Histological Study

**DOI:** 10.3390/pharmaceutics13040540

**Published:** 2021-04-13

**Authors:** Rodrigo Boscariol, Érika A. Caetano, Erica C. Silva, Thais J. Oliveira, Raquel M. Rosa-Castro, Marta M. D. C. Vila, Victor M. Balcão

**Affiliations:** 1 PhageLab—Laboratory of Biofilms and Bacteriophages, University of Sorocaba, Sorocaba 18023-000, SP, Brazil; rodrigoboscariol@yahoo.com.br (R.B.); erikacaetano01@hotmail.com (É.A.C.); erica.silva@edu.uniso.br (E.C.S.); oliveira.thaisjardim@gmail.com (T.J.O.); raquel.rosa@prof.uniso.br (R.M.R.-C.); marta.vila@prof.uniso.br (M.M.D.C.V.); 2 Department of Biology and CESAM, University of Aveiro, Campus Universitário de Santiago, P-3810-193 Aveiro, Portugal

**Keywords:** choline geranate deep eutectic solvent, transdermal permeation, histological changes

## Abstract

In the present research work, we addressed the changes in skin by which deep eutectic solvents (DES) enhanced transdermal permeation of bioactive compounds and propose a rationale for this mechanism. Several studies showed that these unusual liquids were ideal solvents for transdermal delivery of biomolecules, but to date, no histological studies relating the action of DES to changes in the structure of the outer skin barrier have been reported. In the research effort described herein, we presented an in-depth analysis of the changes induced in the skin by choline geranate DES, a compound with proven capabilities of enhancing transdermal permeation without deleterious impacts on the cells. The results obtained showed that a low percentage of DES acted as a transient disruptor of the skin structure, facilitating the passage of bioactive compounds dissolved in it.

## 1. Introduction

The transdermal permeation route received increased attention in recent years due to numerous advantages over the oral and injectable routes of drug administration [1]. Hence, transdermal permeation became an appealing route for the delivery of bioactive macromolecules [2,3]. The main advantages of the transdermal route encompassed the possibility of controlled release over time, elimination of drug degradation in the gastrointestinal tract (GIT) with the enhancement of therapeutic efficacy, and strikingly painless administration [4,5].

The skin is the largest organ in the human body, being a heterogeneous multilayer tissue that covers a surface area of ca. 2 m^2^ and receiving approximately one-third of the blood circulation in the body [5], whose primary function is to protect the body from the external environment by functioning as an effective protective barrier to absorption of exogenous molecules. Such barrier characteristics of the skin are largely due to its composition, making it virtually impenetrable to exogenous large and hydrophilic molecules [1,6,7].

The outermost layer of the skin, termed *stratum corneum*, is responsible for such impermeability being constituted by an association between keratinocytes and lipids [4,6]. A unique class of ceramide lipids is essential for the natural barrier property of the skin, containing linoleic acid (C18:2) esterified to the w-hydroxyl group of a very-long-chain fatty acid linked itself at the N-acyl moiety to sphingosine (C18:1). These lipids, specific to the epidermis, are termed EOS to describe their chemical composition [7,8]: esterified omega-hydroxyacyl-sphingosine. The EOS lipid family integrates a group of structural analogs possessing different carbon chain lengths (viz. C30–C36) and the number of insaturations in the amide-linked very-long-chain fatty acids. The EOS lipids are distributed within the *stratum corneum* alongside cholesterol and saturated fatty acids [8,9]: being organized into trilaminar units that surround corneocytes, the flattened dead cells containing keratin in the outermost layer of the skin. The extremely long fatty acid chain of EOS is essential for the barrier function of the skin. Neutron diffraction studies on *stratum corneum* lipid model systems revealed that one EOS molecule stretches from one lipid membrane layer and penetrates an adjacent layer [7,8,9,10,11,12], hence helping to tighten neighboring bilayers densely together and concomitantly decrease water diffusion. The exact lipid organization of the *stratum corneum* still remains, however, poorly understood due to the difficulty in studying an intact internal structure in native skin tissues in vivo. The skin is, therefore, a formidable barrier that protects nearly the entire human body.

Hence, the topical administration of many drugs requires appropriate technology for attaining successful transdermal drug delivery [13]. In order to overcome such formidable obstacles and to allow the non-invasive delivery of bioactive macromolecules via transdermal permeation, various approaches to weaken the *stratum corneum* have been tested over time, including absorption promoters, iontophoresis, sonophoresis, and micro-needles [4,6].

Absorption promoters increased the cutaneous permeability by increasing either the partition coefficient of the bioactive macromolecule or the thermodynamic action thereof, by modifying the composition of the *stratum corneum* via breaking the lipid structure [6,14] or by creating hydrophilic pores and/or establishing a drug reservoir in the *stratum corneum* itself [15]. In addition, permeation enhancers should exhibit the following characteristics: biocompatibility; nontoxicity; compatibility with the drug being administered; absence of any adverse pharmacological activity inside the body; not be expensive; and be devoid of color, odor, and taste [16]. Recently, ionic liquids (ILs) and deep eutectic solvents (DES) were used for this purpose with success [15,17,18,19,20,21].

ILs and DES are an interesting group of chemical compounds composed exclusively of ions that have melting points below 100 °C, low to negligible vapor pressure at room temperature, extensive and varying solubility profiles, high thermal stability, non-flammability, adaptable polarity, inert chemical profiles, variable viscosities, and many others properties [22,23]. Ionic liquids and their deep eutectic solvents are highly viscous materials made up exclusively of ions, exhibiting melting temperatures below the boiling point of water at atmospheric pressure, that are able to solubilize amphiphilic molecules [22,24]. By definition, the behavior of ILs is dominated by ionic interactions, whereas DES exhibit a strong contribution to hydrogen bonding [22,24]. Choline-based ILs with amino acids are amongst the mainly used ILs in pharmaceutical application studies [20,21]. Recently, choline geranate DES was reported to exhibit virtually nil toxicity to both epithelial cells and skin while greatly enhancing the transdermal delivery of bioactive moieties [17,18,19,25,26]. The transdermal delivery potentiation and the virtual absence of cytotoxicity of this DES are directly related to their chemical properties and constituent cations and anions [25,26].

ILs and DES are able to solubilize amphiphilic molecules and are, therefore, ideal solvents for the transdermal delivery of macromolecules. The molecules of these highly viscous fluids are hypothesized to slip around and through the fatty compounds that make up the skin cells, creating small transient openings through which bioactive molecules can permeate [22]. However, to date, no definitive proof of this putative mechanism has been put forward. Human skin permeability has been reported to vary (both inter- and intra-individual), and, therefore, pigskin is commonly used as an alternative model to human skin in percutaneous absorption studies aiming at developing transdermal formulations [27].

In this sense, the major goal of the research work described herein was to try to uncover the mechanism by which DES, and particularly choline geranate, enhance transdermal permeation using porcine ear skin as a skin model and curcumin as the bioactive substance to be permeated.

## 2. Materials and Methods

### 2.1. Materials

#### 2.1.1. Cell Lineages

The 3T3 and HaCaT (immortalized human keratinocytes) cell lines used in the genotoxicity (Comet™) and MTT assays were purchased from Sigma-Aldrich (St. Louis, MO, USA). The cells were maintained at 37 °C according to the procedure described by Rocha et al. [28].

#### 2.1.2. Porcine Ear Skin

Domestic pig ears were obtained from a local market in the region of Sorocaba (São Paulo, Brazil) and, from these, the skin was excised and used in the transdermal permeation assays.

#### 2.1.3. Chemicals

Tap water was purified in a Master System All (model MS2000, Gehaka, São Paulo, Brazil) to a final resistivity of ca. 18.18 MΩ.cm and conductivity of 0.05 µS.cm^−1^. Geranic acid (85% stabilized; ref. W412101-1KG-K) and choline bicarbonate (ref. C7519-500ML) were purchased from Sigma-Aldrich (St. Louis, MO, USA). HPLC-grade methanol (LiChrosolv^®^, CAS-No: 67-56-1) was purchased from Merck (Darmstadt, Germany).

### 2.2. Experimental Procedures

#### 2.2.1. Synthesis and Characterization of DES

##### Synthesis of Choline Geranate Deep Eutectic Solvent (CG-DES)

CG-DES (1:2) were prepared following the methodology described in detail elsewhere [17,19]. Briefly, a ratio of choline bicarbonate to geranic acid of 1:2 was used to synthesize CG-DES. To a 250 mL round bottom flask, 18.72 g (or 0.1133 mol) of pure choline bicarbonate were added, followed by the addition of 39.58 g (or 0.2350 mol) of pure geranic acid (in excess, to ensure a maximum extent of the reaction). Twenty milliliters of methanol were then added to the mixture in order to reduce the viscosity, and magnetic stirring proceeded overnight at room temperature in an open system fashion until CO_2_ production ceased. The solvent was subsequently removed (60 °C during ca. 20 min) using a rotary evaporator, the resulting CG-DES were transferred into a 50 mL Falcon tube, the headspace of which was flushed with nitrogen, and the tube was immediately capped and duly sealed with Parafilm™ (Bemis Flexible Packaging, Neenah, WI, USA).

##### Assessment of the Cytotoxicity Potential of Plain CG-DES via Mitochondrial Activity (MTT) Assay

Evaluation of the cytotoxicity potential of plain CG-DES to HaCaT (immortalized human keratinocyte) cell lines (assessment of cellular viability) was performed using the MTT (3-(4,5-dimethylthiazol-2-yl)-2,5-diphenyltetrazolium bromide) assay, following the procedure described in detail elsewhere [17,18,19,28].

##### Assessment of the Genotoxicity Potential of Plain CG-DES, via the Comet™ Assay

Cell lineage 3T3 was placed in contact with the sample (250 µL CG-DES were diluted in 10 mL ultrapure water and, from the resulting solution, 300 µL were used for the test), with the negative being a plain DMEM (Dulbecco’s Modified Eagle’s medium, Gibco Life Technologies, São Paulo/SP, Brazil) medium in contact with the 3T3 cells, for a period of 1 h. Tests were carried out in triplicate following the procedure described in detail elsewhere [17,28].

#### 2.2.2. The Effect of DES on Skin Permeability

##### Porcine Ear Skin Preparation

The ear skin was prepared according to the procedure described by Salerno et al. [29], with slight modifications [18,19]: the skin was excised from domestic pig ears (4 to 6 months old, obtained from a local market in the region of Sorocaba (São Paulo, Brazil)), and the ears were thoroughly cleaned under tap water immediately after excision. Any hair from the external region of the ears was duly removed, the skin was carefully separated from the cartilage using a scalpel, and subcutaneous adipose tissue was carefully removed so as to produce a uniform thickness of 1 mm (controlled with a caliper rule). Although the porcine ear skin presents some anatomical differences relative to the human skin, there is a relative uniformity of the samples and ease of dissection and separation of the subcutaneous tissue [13,30]. After being dried with a paper tissue, the skin discs were immediately mounted on the Franz vertical diffusion cells [31,32].

##### Preparation of DES Suspensions

Suspensions of CG-DES in saline solution (0.9% NaCl, *w*/*w*) were prepared at different CG-DES percentages (*w*/*v*): 0.5%, 1.0%, 1.5%, 2.0%, and 2.5%.

##### In Vitro Skin Permeation Testing

**Figure 1:**
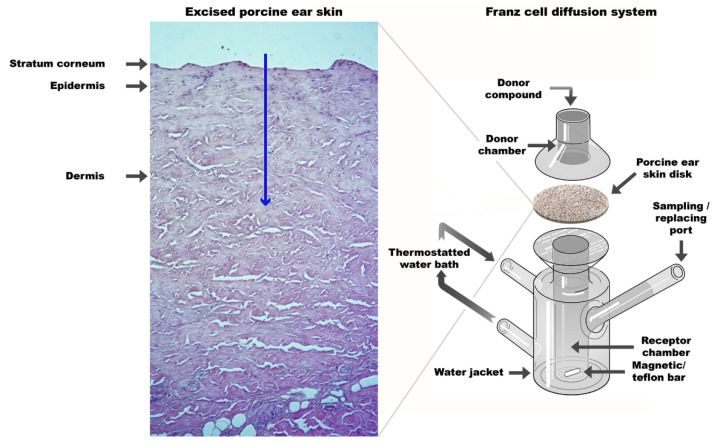
Schematic diagram of the static Franz diffusion cell used in the in vitro permeation tests with CG-DES showing the pathway for drug permeation (blue arrow).

The non-invasive effect of CG-DES in enhancing skin permeation was studied in a DHC-6T Transdermal System from Logan Instruments Corporation (Somerset, NJ, USA), using thawed porcine ear skin discs (0.5 mm thick × 30 mm ϕ_ext_). The porcine ear skin discs were then clamped on top of the Franz diffusion cell (with a 15 mm ϕ_ext_ central hole, contacting the receptor fluid beneath; Figure 1). On top of these porcine ear skin discs, a cavity with ϕ_ext_ = 30 mm was carefully filled with 2 mL ionic liquid suspension (integrating different volume percentages of CG-DES, containing or not curcumin) eliminating any air bubbles. To the receptacle of the Franz diffusion cell (possessing a small cylindrical Teflon-coated magnetic stirring bar) beneath the porcine ear skin disc, 8 mL of saline solution (0.9% NaCl, *w*/*w*) buffered to pH 7.0 were carefully poured until touching the lower surface of the skin ensuring the absence of air bubbles and stretching the skin so as to minimize the presence of furrows. This compartment was maintained at 32 ± 3 °C for 30 min. The CG-DES suspension (with or without curcumin) was allowed to contact the upper part of the porcine ear skin discs for a period of 12 h. When evaluating curcumin permeation, 2-mL samples were withdrawn from the receiving fluid beneath the porcine ear skin disk at predetermined time intervals over a timeframe of 6 h, and 2-mL of fresh saline solution (pH 7.0) were duly added so as to reset the volume. Each sample was then assayed spectrophotometrically for curcumin quantification at a wavelength of 421 nm using disposable plastic cuvettes (Kartell, Noviglio MI, Italy) in a UV-Vis Spectrophotometer from Agilent (model Cary 60 UV-Vis, Santa Clara, CA, USA). A calibration curve for curcumin was prepared using solutions of curcumin in buffered saline solution (0.9% NaCl, *w*/*w*, pH 7.0) at several concentrations in the range 0–25 µg/mL: $Abs_{421\;nm}=0.0606\;\times \;\left[curcumin,\;\mu g/mL\right]+0.0029\;\left(r^{2}=0.9983\right)$.

##### Skin Conservation for Histological Preparations

Following the transdermal permeation tests and separation of the section of interest of the skin discs in contact with the suspensions of CG-DES, the tissues were immediately placed in 10% formalin buffer (fixative) in order to prevent solubilization of tissue proteins. The porcine ear skin samples were dehydrated with increasing concentrations of ethanol in order to avoid a pronounced tissue retraction which would cause irreversible cellular structural lesions.

#### 2.2.3. Preparation of Porcine Ear Skin Histological Cuts

The histological analysis of the porcine ear skin discs contacted with different percentages (*v*/*v*) of CG-DES (0.5%, 1.0%, 1.5%, 2.0%, and 2.5%) was performed after each porcine ear skin disc had been fixed and processed. Tissue fixation was carried out in buffered formalin for 48 h time, after which the samples were washed overnight in running water, and the tissues were then stored in ethanol at 70% (*v*/*v*). Subsequently, the tissues were treated in an automatic tissue processor (from O Patologista^®^, model PT12, Guarulhos SP, Brazil), and the pieces were embedded in histological paraffin according to the sequence [33]: (i) dehydration (passage in ethanol at 70% (*v*/*v*); 50 min in ethanol at 90% (*v*/*v*); 150 min in ethanol at 100% (*v*/*v*)), (ii) clarification with xylol during 120 min, and (iii) impregnation with paraffin during 120 min. The histological cuts of the porcine ear skin samples, with a thickness of four micrometers, were prepared in a rotary-manual microtome (from O Patologista^®^, model MR 2014, Guarulhos, SP, Brazil) and then stained via hematoxylin and eosin staining techniques: (i) dewaxing with xylol for 30 min, (ii) hydration with ethanol at 100% (*v*/*v*) for 20 s, (iii) hydration with ethanol at 95% (*v*/*v*) for 10 s, (iv) hydration with ethanol at 70% (*v*/*v*) for 10 s, (v) washing with water for 2 s, (vi) staining with hematoxylin for 6 min, (vii) washing with running water for 3 min, (viii) staining with eosin for 2 min, (ix) washing with water for 2 s, (x) dehydration with ethanol at 70% (*v*/*v*) for 10 min, (xi) dehydration with ethanol at 100% (*v*/*v*) for 20 min, (xii) fixing the dye and preserving the material with xylol for 1 s, and (xiii) fixing the dye and preserving the material with xylol until the slide was assembled, and, finally, (xiv) embedded with histological resin [33,34]. The analysis of the histological sections was performed qualitatively with the aid of an optical microscope coupled to a digital camera (Biological Microscope, Model Axio Lab. A1, Zeiss^®^, Oberkochen, Germany).

## 3. Results

In the present research work, we proposed a putative mechanistic model for the action of CG-DES in enhancing transdermal permeation of curcumin. Since intermolecular interactions between the DES, curcumin, and skin components were also likely to play a role in the transdermal delivery of curcumin, one also performed the photomicrographical study of the histological section of the porcine ear skin after the transdermal permeation of curcumin using CG-DES at 2.0% (*w*/*v*), and compared the results with those of the blank experiments, i.e., without permeating any substance, and also with porcine ear skin contacted with only saline solution (0.9% NaCl, *w*/*w*).

**Figure 2:**
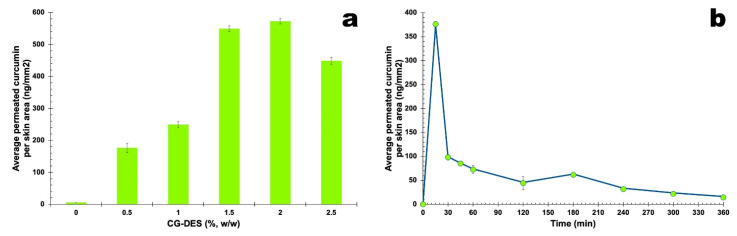
Results gathered from transdermal permeation assays of curcumin aided by CG-DES, as average permeated curcumin ($\mathrm{ng}/{\mathrm{mm}}_{\mathrm{skin}}^{2}$), for several CG-DES concentrations (**a**) and during a 6 h assay using the best CG-DES concentration (viz. 2%, *w*/*w*) (**b**). All values represent the mean of three experiments and the error bars represent the standard deviations.

Figure 2a displays the results obtained for the maximum average permeated curcumin for different amounts of CG-DES (%, *w*/*w*), allowing us to determine the optimum concentration of CG-DES to be used in the transdermal permeation assays, whereas Figure 2b displays the results of the transdermal permeation assays of curcumin as a function of time for the optimum CG-DES concentration.

**Figure 3:**
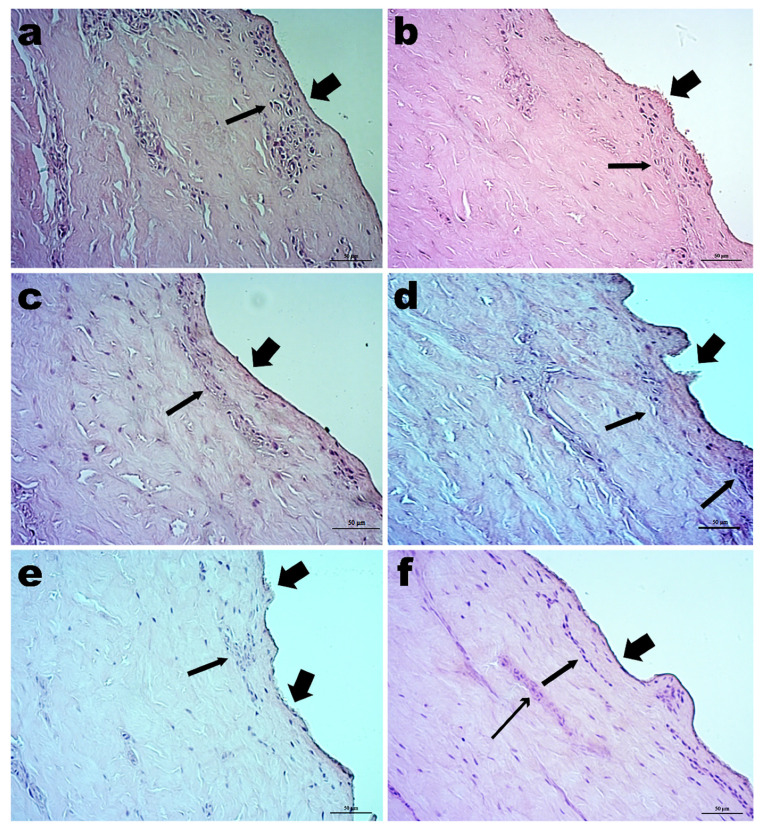
Photomicrographs (×200 magnification) of (**a**) the histological sections of plain porcine ear skin, and of porcine ear skin following contact with (**b**) 0.5% CG-DES, (**c**) 1.0% CG-DES, (**d**) 1.5% CG-DES, (**e**) 2.0% CG-DES, and (**f**) 2.5% CG-DES. All sections were stained with hematoxylin and eosin. For the legend of inserted (thick, thin, and skinny) arrows, please refer to the Discussion section.

The results of the histological analysis performed on the porcine ear skin following the contact with CG-DES and the staining with hematoxylin and eosin are depicted in Figure 3 in the form of photomicrographs with ×200 magnification.

In Figure 3, it is possible to observe that no significant morphological changes were produced, following contact of the skin with CG-DES. However, possible breakdowns in the *stratum corneum* were noticeable depending on the concentration of CG-DES used. The cell nuclei of the basal layer were most evident in the photomicrographs since the histological sections were performed, favoring visualization between the epidermis and the dermis. It was worth noting that areas of cartilage and adipose tissue were excluded from these histological cuts.

**Figure 4:**
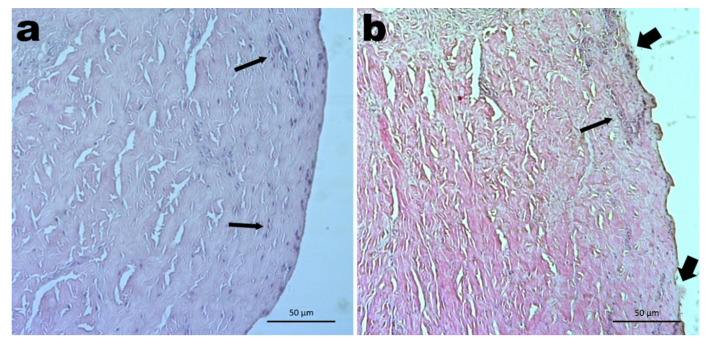
Photomicrographs (×200 magnification) of (**a**) the histological section of porcine ear skin contacted with only saline solution (0.9% NaCl, *w*/*w*) and (**b**) the histological section of the porcine ear skin after transdermal permeation of curcumin using CG-DES at 2.0% (*w*/*v*). All sections were stained with hematoxylin and eosin. For the legend of inserted (thick, thin, and skinny) arrows, please refer to the Discussion section.

Figure 4 displays the histological cuts of porcine ear skin exposed to plain saline solution (0.9% NaCl, *w*/*w*; Figure 4a) and after transdermal permeation of curcumin using CG-DES at 2.0% (*w*/*v*; Figure 4b).

CG-DES promoted a great enhancement in the transdermal permeation of curcumin, the bioactive molecule used as a model in this work (Figure 2a). These results were in close agreement with previous findings from our research group, namely in the transdermal permeation of insulin [17] and in the transdermal permeation of bacteriophage particles to treat skin infections (either in the deeper layers of the skin and in the more superficial ones) [18,19]. CG-DES were able to solubilize amphiphilic molecules and were, therefore, an ideal solvent for the transdermal delivery of macromolecules such as the aforementioned ones.

Monti et al. [15] suggested that ionic liquids could affect transdermal permeation by changing the electric properties of the *stratum corneum* and favoring the transfer of the drug into the deeper layers of the skin; however, the exact mechanism by which this happens remains poorly understood, and, to date, no definitive proof of this putative rationale has been put forward.

**Figure 5:**
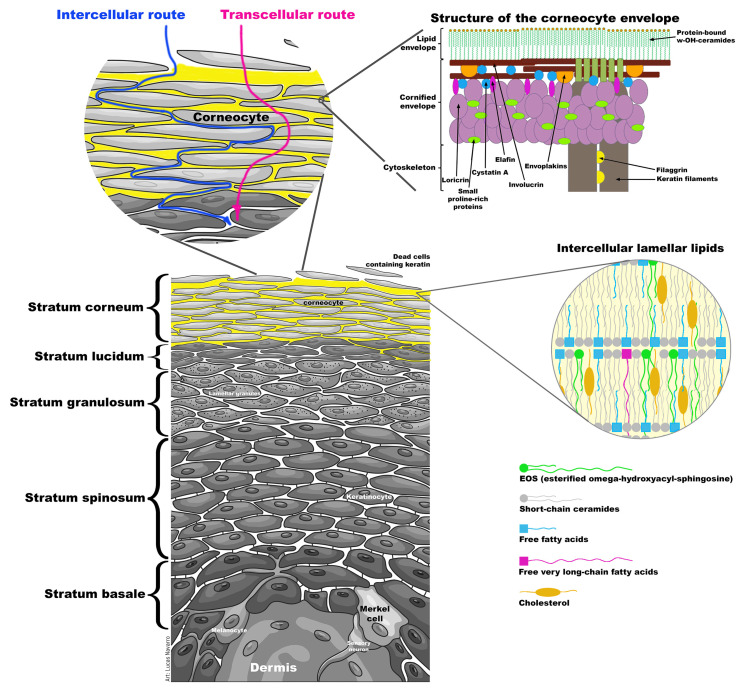
Schematic representation of the skin displaying the skin barrier (i.e., the *stratum corneum*, consisting of corneocytes surrounded by a multilamellar lipid matrix of ceramides, free fatty acids, and cholesterol densely packed in an approximately 1:1:1 molar ratio), the structure of the corneocyte envelope, and two putative transdermal permeation routes potentiated by CG-DES. Parts of Figure 5 were adapted from [35,36], Cayman Chemical, 2020 and Martin-Luther-Universität Halle-Wittenberg, 2018.

Figure 5 depicts a schematic representation of the skin, displaying the skin barrier (i.e., the *stratum corneum*) and two putative transdermal permeation routes potentiated by CG-DES.

One of the many important biological functions of ceramides is to maintain skin moisture, with a unique class of ceramides being indispensable for maintaining the natural barrier of the skin. Such class of ceramide lipids integrate linoleic acid, the most abundant essential fatty acid present in the epidermis, esterified to the w-hydroxyl group of a very-long-chain fatty acid linked at the N-acyl moiety to sphingosine. A good understanding of the structure of the skin barrier, the *stratum corneum*, is therefore essential if one intends to postulate a rationale for the mechanism of action of ionic liquids as permeation enhancers. In Figure 5, two putative routes for permeation of bioactive molecules potentiated by CG-DES have been put forward.

## 4. Discussion

Based on the many reported uses of ILs and DES as permeation enhancers, there was a clear interest in using these substances in transdermal drug delivery, but the associated permeation mechanism remained essentially unclear. ILs were shown to enhance the transdermal transport, either transcellularly and paracellularly; however, there were indications that ILs bypass the barrier properties of the *stratum corneum* employing mechanisms such as disruption of cellular integrity, fluidization, creation of diffusional pathways, and extraction of lipid components in the *stratum corneum* [23].

Curcumin is a phytopolylphenol pigment isolated from the plant *Curcuma longa*, commonly known as turmeric, a natural product with therapeutic potential for skin diseases such as (but not limited to) psoriasis and cancer [37,38,39], but it has quite low aqueous solubility, which contributes to the low absorption of curcumin from the GIT and the low oral bioavailability due to the rapid first-pass metabolism, which limits its usefulness as an oral drug but makes it an interesting molecule for administration through the skin [40,41,42]. In this context, the transdermal route was an important strategy for the administration of bioactive macromolecules since it avoids the first-pass biotransformation and allows the use of self-administered pharmaceutical forms, with concomitant improvement of patient compliance [43,44]. However, due to both the excellent barrier function of the *stratum corneum* and the relatively high lipophilicity and biochemical/structural degradation of curcumin, skin delivery of curcumin was challenging. Hence, it was chosen as a model biomacromolecule for the study.

In the research work described herein, the performance of a transdermal permeation enhancer (viz. CG-DES) was evaluated via in vitro permeation testing (IVPT) using porcine ear skin as the skin model. The animal models used to replace human skin in transdermal permeation studies are domestic pigs, rats, mice, guinea pigs, and snakes. However, porcine ear skin showed results comparable to those of normal human skin [45].

The synthesized CG-DES showed virtually nil genotoxic effects in 3T3 cells [17], closely agreeing with the lack of significant cytotoxic effects promoted by CG-DES, and, overall, these results were in clear agreement with results published elsewhere [19,21,45].

In the photomicrograph of Figure 3a, one can observe the histological section of plain porcine ear skin (without any contact with CG-DES) following staining with hematoxylin and eosin, evidencing the *stratum corneum* (thick arrow) without any flaking and the basal layer (thin arrow) formed by cuboid or cylindrical cells (stem-cells). In this *stratum*, in addition to keratinocytes, Merkel cells and melanocytes (cell types not identifiable in routine preparations) were also present. Right after the basal layer, one could observe the compact dermis consisting of dense connective tissue not modeled with collagen fibers, stained by eosin (pink to red), thick, and oriented in several directions. The *stratum corneum* did not present changes in either its structure and disposition of the epidermis, making it possible to observe the keratin layers with an intact structure and appearance (thick arrow). The basal lamina (thin arrow) had no nuclear or eosinophilic cytoplasm changes in its cuboid or cylindrical cells, and the dermis showed no changes in the amount and disposition of collagen fibers stained by eosin.

The photomicrograph in Figure 3b shows the histological cut of the porcine ear skin after exposure to 0.5% (*w*/*w*) CG-DES for 12 h suggesting a possible deconstruction of the corneal layer of the epidermis (thick arrow); however, not being observed in the basal layer nuclear or cytoplasmic changes in its cuboid or cylindrical cells (thin arrow), nor changes in the amount and disposition of collagen fibers stained by eosin in the dermal layer.

The photomicrograph in Figure 3c shows the histological section of the porcine ear skin exposed to 1.0% (*w*/*w*) CG-DES. The thick arrow indicated the *stratum corneum* without changes either in its structure or in the disposition of the epidermis, and the basal layer was also shown without nuclear changes or changes in the eosinophilic cytoplasm (thin arrow). The dermis layer showed no changes in the amount and disposition of collagen fibers, stained by eosin.

The photomicrograph in Figure 3d shows the histological cut of the porcine ear skin exposed to 1.5% (*w*/*w*) CG-DES. The contact with this concentration of CG-DES suggested a possible deconstruction of the *stratum corneum* of the skin and the layers of keratin (thick arrow), with the basal layer showing no nuclear changes and maintaining preserved the eosinophilic cytoplasm (thin arrows). The compact dermis showed no changes in the amount and disposition of collagen fibers.

The photomicrograph in Figure 3e shows the histological cut of the porcine ear skin exposed to 2.0% (*w*/*w*) CG-DES. This photomicrograph indicated two sites of possible deconstruction of the *stratum corneum* of the epidermis disorganizing the keratin layers (thick arrows). The basal lamina was, however, preserved (thin arrow), with oval nuclei and eosinophilic cytoplasm showing its preserved cuboid or cylindrical cells. The dermis still showed no changes in the amount and disposition of collagen fibers.

The photomicrograph in Figure 3f shows the histological section of the porcine ear skin exposed to 2.5% (*w*/*w*) CG-DES. After the contact with this concentration of CG-DES, the *stratum corneum* of the epidermis did not show flaking, and the keratin layers maintained their structure, and the appearance stayed intact (thick arrow). The basal lamina (thin arrow) presented no nuclear or cytoplasmic changes maintaining preserved its cuboid or cylindrical cells. The dermis continued to show no changes in the amount of collagen fibers showing only slight compaction of these fibers (skinny arrow).

After analyzing all the histological sections shown in Figure 3, one may conclude that the contact of the porcine ear skin with various concentrations of CG-DES did not cause significant morphological changes in the skin. However, possible deconstructions were observed (although to a limited extent) in the *stratum corneum* after contact with some concentrations of CG-DES (photomicrographs b, d, and e in Figure 3). The basal layer nuclei were the most evident ones in all histological sections since the histological preparations were performed favoring visualization between the epidermis and the dermis. It was also worth noting that areas of cartilage and adipose tissue were disregarded from all histological cuts.

In Figure 2a, one can observe that the maximum average permeated curcumin for different amounts of CG-DES (%, *w*/*w*) were higher for 2% (*w*/*w*) CG-DES, and this amount of CG-DES allowed the transdermal permeation of a reasonable amount of curcumin (viz. ca. 375 ng_curcumin_/mm^2^_skin_) in only 15 min of contact with the skin, followed by a steady transdermal permeation of curcumin between 75 ng_curcumin_/mm^2^_skin_ and 25 ng_curcumin_/mm^2^_skin_ up to the 6 h timeframe of the assay (Figure 2b). Despite promoting a slight deconstruction of the *stratum corneum*, as apparent from inspecting the histological cut in the photomicrograph of Figure 3e, this amount of CG-DES were highly effective in enhancing the transdermal permeation of bioactive macromolecules [17,18,19] without promoting major morphological changes in the skin, as can be observed in Figure 3.

The photomicrographs in Figure 4 show the histological cuts of porcine ear skin exposed to the plain saline solution (0.9% NaCl, *w*/*w*; Figure 4a) and after transdermal permeation of curcumin using CG-DES at 2.0% (*w*/*v*; Figure 4b). In the photomicrograph of porcine ear skin exposed to plain saline solution, no deconstructions or flaking of the *stratum corneum* could be observed, and the basal layer (thin arrows) formed by cuboid or cylindrical cells (stem-cells) were well observed. In the photomicrograph of porcine ear skin after transdermal permeation of curcumin using CG-DES at 2.0% (*w*/*v*; Figure 4b), two sites of possible deconstruction of the *stratum corneum* of the epidermis were apparent, with disorganization of the keratin layers (thick arrows). The basal lamina was maintained, however, preserved (thin arrow), with oval nuclei and eosinophilic cytoplasm, also showing its preserved cuboid or cylindrical cells, although with slight compaction. These results were, in fact, quite similar to those of the blank experiments (i.e., without permeating curcumin, see photomicrograph in Figure 3e) using the same weight percentage of CG-DES.

As apparent from the histological cuts displayed in Figure 3, the skin maintained its morphological structure virtually intact upon contacting with CG-DES, and a possible explanation for the permeation enhancing properties of CG-DES might be that this DES promoted transdermal permeation through the skin layers via the two putative routes illustrated in Figure 5. According to Chantasart and Kevin [46], increased permeation depended strongly on the lipophilicities of the permeation enhancers. The mechanism of the action of the permeation enhancers was related to the fluidization of the intercellular lipids in the *stratum corneum*, supporting the hypothesis that the site of action of the permeation enhancer was in the semi-polar region of the intercellular lipid domain in the *stratum corneum*, promoting drug penetration across the *stratum corneum* lipoidal pathway (intercellular route, Figure 5). However, it seemed that the transdermal permeation promoted by CG-DES also occurred in a more direct way, viz the transcellular route (Figure 5).

Islam et al. [47] developed a series of microemulsions with acyclovir and ionic liquid (choline oleate) to evaluate in vitro the permeation of acyclovir through the skin. The results obtained by those researchers revealed that the acyclovir permeation was intensified by using the ionic liquid. They observed by FTIR analyses that the enhanced drug permeation occurred due to a reduction of the skin barrier function via modification and disruption of the regular arrangement of the corneocytes of the *stratum corneum*, which corroborated the results presented herein and confirmed the putative transdermal permeation routes potentiated by CG-DES that were put forward.

The molecules of CG-DES were therefore hypothesized to slip around (intercellular route, through the intercellular lamellar lipids, see Figure 5) the corneocytes that made up the *stratum corneum,* and through (transcellular route) both the intercellular lamellar lipids and the fatty compounds that made up the plasma membrane of skin cells (viz, the corneocyte lipid envelope, see Figure 5), creating small transient openings through which the bioactive molecules could permeate [22].

## 5. Conclusions

In this research effort, the effect of CG-DES on the skin was studied following abounding experimental evidence that this DES promoted transdermal permeation of bioactive macromolecules. The results of this study clearly showed that the use of CG-DES highly enhanced transdermal permeation, and a rationale for the mechanism of action by which CG-DES permeabilized the skin was postulated based on an in vitro skin histological study. The molecules of CG-DES were hypothesized to slip around (intercellular route, through the intercellular lamellar lipids) and through (transcellular route) the fatty compounds that made up the corneocyte lipid envelope creating small transient openings through which bioactive molecules could permeate. The use of CG-DES in topical formulations was proved by our research group to greatly increase the transdermal permeation of curcumin (which will enable advances in the application of this biomacromolecule to treat chronic skin diseases), insulin (which will enable the development of non-invasive systems for insulin administration), and bacteriophage particles (enabling the treatment of localized bacterial infections in the deeper layers of the skin). The system developed will also allow the non-invasive delivery of a wide range of bioactive macromolecules with poor water solubility in an easy fashion, with a high potential for translation into real-world applications with clear benefits to patients.

## Data Availability

The data presented in this study is contained within the article.
